# Kyste de l'ovaire et grossesse

**DOI:** 10.11604/pamj.2014.17.99.3206

**Published:** 2014-02-08

**Authors:** Nadia Khoummane, Sabah El Amrani

**Affiliations:** 1 Université Mohamed V, Service de Gynécologie Obstétrique, de Cancérologie et de Grossesses à haut risque, Maternité Souissi, Rabat, Maroc

**Keywords:** Kyste ovairien, grossesse, césarienne, ovarian cyst, pregnancy, caesarean

## Image en medicine

La découverte d'un kyste de l'ovaire pendant la grossesse est une situation de plus en plus fréquente en raison de la pratique systématique de l’échographie dans le suivi prénatal. L'incidence de survenue de kyste ovarien pendant la grossesse est estimée à environ 1%. Le diagnostic du kyste de l'ovaire durant la grossesse pose des problème de prise ne charge thérapeutique selon le terme pendant lequel a été découvert le kyste. Les complications les plus fréquentes sont la rupture du kyste et la torsion. Le traitement chirurgical pendant la grossesse comporte des risques foetaux et maternels. La laparoscopie est actuelement le gold standard dans le traitement de kyste de l'ovaire découvert pendant la grossesse. Nous rapportons ici le cas d'une parturiente non suivie qui s'est présentée à la maternité en travail et chez qui une échographie en salle de travail a révélé un kyste de l'ovaire gauche faisant un obstacle praevia, une césarienne fut indiquée en urgence, après extraction du nouveau-né et hystérorraphie une kystectomie a été réalisée. Cette césarienne aurait pu etre évitée si la patiente avait consulté auparavant et bénéficié d'une laparoscopie permettant l'exérèse chirurgicale du kyste ainsi que de tenter un accouchement par voie basse.

**Figure 1:**
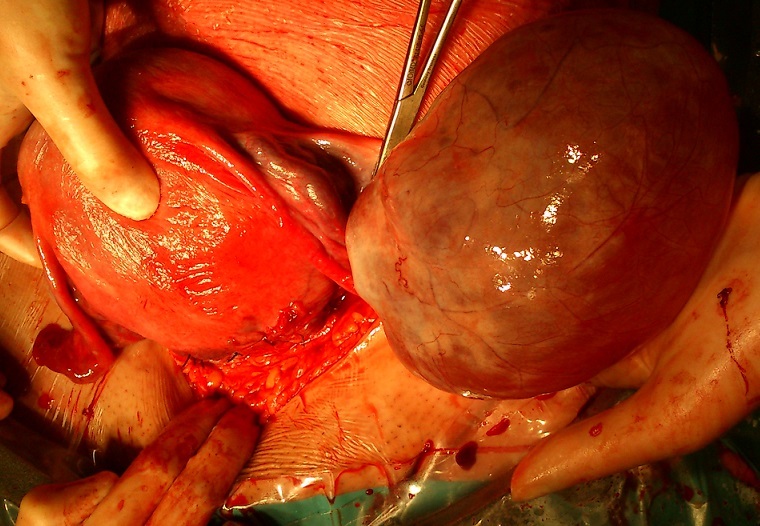
Kyste ovarien de 11 cm réalisant un obstacle praevia conduisant à la pratique d'une césarienne

